# Person identification from EEG using various machine learning techniques with inter-hemispheric amplitude ratio

**DOI:** 10.1371/journal.pone.0238872

**Published:** 2020-09-11

**Authors:** Isuru Jayarathne, Michael Cohen, Senaka Amarakeerthi

**Affiliations:** 1 Spatial Media Group, University of Aizu, Aizu-Wakamatsu, Fukushima, Japan; 2 Dept. of Information and Communication Technology, University of Sri Jayewardenepura, Gangodawila, Nugegoda, Sri Lanka; Universita degli Studi di Catania, ITALY

## Abstract

Association between electroencephalography (EEG) and individually personal information is being explored by the scientific community. Though person identification using EEG is an attraction among researchers, the complexity of sensing limits using such technologies in real-world applications. In this research, the challenge has been addressed by reducing the complexity of the brain signal acquisition and analysis processes. This was achieved by reducing the number of electrodes, simplifying the critical task without compromising accuracy. Event-related potentials (ERP), a.k.a. time-locked stimulation, was used to collect data from each subject’s head. Following a relaxation period, each subject was visually presented a random four-digit number and then asked to think of it for 10 seconds. Fifteen trials were conducted with each subject with relaxation and visual stimulation phases preceding each mental recall segment. We introduce a novel derived feature, dubbed Inter-Hemispheric Amplitude Ratio (IHAR), which expresses the ratio of amplitudes of laterally corresponding electrode pairs. The feature was extracted after expanding the training set using signal augmentation techniques and tested with several machine learning (ML) algorithms, including Linear Discriminant Analysis (LDA), Support Vector Machine (SVM), and k-Nearest Neighbor (kNN). Most of the ML algorithms showed 100% accuracy with 14 electrodes, and according to our results, perfect accuracy can also be achieved using fewer electrodes. However, AF3, AF4, F7, and F8 electrode combination with kNN classifier which yielded 99.0±0.8% testing accuracy is the best for person identification to maintain both user-friendliness and performance. Surprisingly, the relaxation phase manifested the highest accuracy of the three phases.

## Introduction

A person identification system verifies the identity of a given individual from a set of people. In contrast, authentication uses different classification methods (such as one-class classification, template matching, and score level fusion) to confirm identity. Both identification and authentication have the same pre-processing and feature-extraction steps. An identification process is often conducted in police departments using records or visual information of arrested criminals as biometrics. Even though potential applications in this domain are fewer than those for authentication, the process can be enhanced to create an authentication system. Currently, there are several strategies for both cases, including knowledge (such as of a passcode), possession (such as of an ID card), and biometric traits. Biometric-based techniques use biological or physiological attributes such as fingerprint, palm-print, iris, and voice to identify someone. Biometrics are usually more convenient and intimately personal compared to other strategies.

A brain-computer interface (BCI) or neural-control interface (NCI) represents direct communication between an external device and an enhanced or exposed brain. Besides functional magnetic resonance imaging (fMRI) and positron emission tomography (PET), which observe changes of blood flow, other methods can track electrical activity, such as electroencephalography (EEG) and magnetoencephalography (MEG). EEG is a non-invasive, electrophysiological monitoring method to record electrical activity of a human brain. International standards have been established regarding placement of electrodes over the human scalp with different resolutions. The 10-20 system is the most popular for low-resolution capture, and the 10-5 system, which allows more than 300 electrodes, has highest resolution. Since EEG is innocuous, medically safe, it has attracted considerable attention from the scientific community for such applications as machine learning, robotics, and health care. This study mainly focuses on person identification using EEG analysis to find unique brain signal patterns.

### 0.1 Related research

Relevance for individually personal information in EEG signals was revealed in the early 1930s [1]. Even though EEG has low spatial resolution compared to fMRI, several studies have shown considerable accuracy in this domain [2–4]. Pozo-Banos et al. [5] reported in a comprehensive review about EEG subject identification that EEG has subject-specific information. Jayarathne et al. [6] and Almehmadi et al. [7] compiled comprehensive surveys of EEG-based access control systems. These surveys reported suitability, state of the art of EEG for person identification, and important parameters which must be adjusted to implement an authentication system with desired security and usability levels. To find unique features of EEG signals, researchers have used different kinds of stimulation, including visually evoked potentials (VEP) [8], auditory stimulation, motor movement, and assigned math problems [9]. Also, devices used to capture EEG signals have various attributes, such as number of electrodes, electrode type (dry or wet), and wired or wireless computer connection. Chen et al. have shown that wet electrodes allow better signal quality than dry ones [10]. Nevertheless, dry electrode headsets provide more user-friendliness. Despite perfect accuracy having been achieved in several studies, most systems are not very convenient or user-friendly.

The brain emits different EEG signal patterns according to stimuli and responses. Researchers have confirmed that as stimulus sets become increasingly complex, distinctiveness of subjects becomes higher [5]. Moreover, visual stimulation is the most promising and popular modality in this field. Poulos et al. [11, 12] tested two different approaches with the same computational geometry classification algorithm without using any specific task. They achieved 91% and 95% accuracy for Auto Regression (AR) type of alpha-band EEG signals, extracting spectral features via Fourier Transform (FT), and 84% maximum accuracy for Learning Vector Quantizer (LVQ) network with AR parameters as features [2]. Singhal et al. [13], Das et al. [8], Palaniappan et al. [14], and Yazdani et al. [15] used VEP as a stimulation when capturing EEG signals, achieving 78%, 94.08%, 94.18%, and 100% accuracy respectively. Most approaches have used temporal domain features and achieved considerable accuracy.

In our approach, a new feature called Inter-Hemispherical Amplitude Ratio (IHAR) is introduced, and features are extracted from various mental task data sets to find uniqueness. A similar technique (inter-hemispheric amplitude relationship) was used by Goldstein et al. to observe differences of REM and NREM sleep patterns of humans, cats, and rabbits [16]. Goldstein’s approach emphasizes differences of REM and NREM stages of EEG signal for easy observation, although a classification technique was not used. Even though various time- and frequency-domain features have been proposed, feature extraction method with low computational complexity is better for embedded system implementations with low computational power budget. Several studies can be found about various asymmetries of the head, concerning such topics as skull asymmetry [17], structural cerebral asymmetry [18], and functional cerebral asymmetry [19]. Functional asymmetry has been analyzed using fMRI, PET [20], and EEG in different domains. Based on functional asymmetry of the brain, IHAR was calculated to find subjective features to distinguish people.

Classification is the most popular supervised learning problem domain in machine learning (ML). This process is concerned with identifying the sub-population to which each given observation belongs, based on a training set of tagged data containing observations whose sub-populations are known. There are various algorithms to solve this kind of problem, including Linear Discrimination Analysis (LDA), Support Vector Machine (SVM), k-Nearest Neighbor (kNN), and Decision Trees. Before training these algorithms, features must be extracted from the raw data. The above-described IHAR feature was extracted and tested with different ML classification algorithms, discussed in next section. Furthermore, we noted the accuracy while reducing the number of channels to increase user-friendliness of EEG capturing devices, because less calibration time is required for minimal electrode setups.

## 1 Methodology

### 1.1 Experimental setup and apparatus

The same dataset used in a previous experiment [21] was used to perform the analysis reported here. Twelve mentally healthy subjects (6 males and 6 females, ages 24 to 45) were recruited for this experiment, including students and academic staff at the Sabaragamuwa University of Sri Lanka. An equal number of males and female subjects were selected to avoid gender bias. The experiment was performed after obtaining written consent. The Sabaragamuwa University of Sri Lanka ethics committee waived the need for ethical approval. Since our focus is to implement an identification system for a small group of users, we decided to conduct this experiment with a limited number of users. However, scalability issues of the proposed approach are discussed in the results and discussion section.

**Fig 1:**
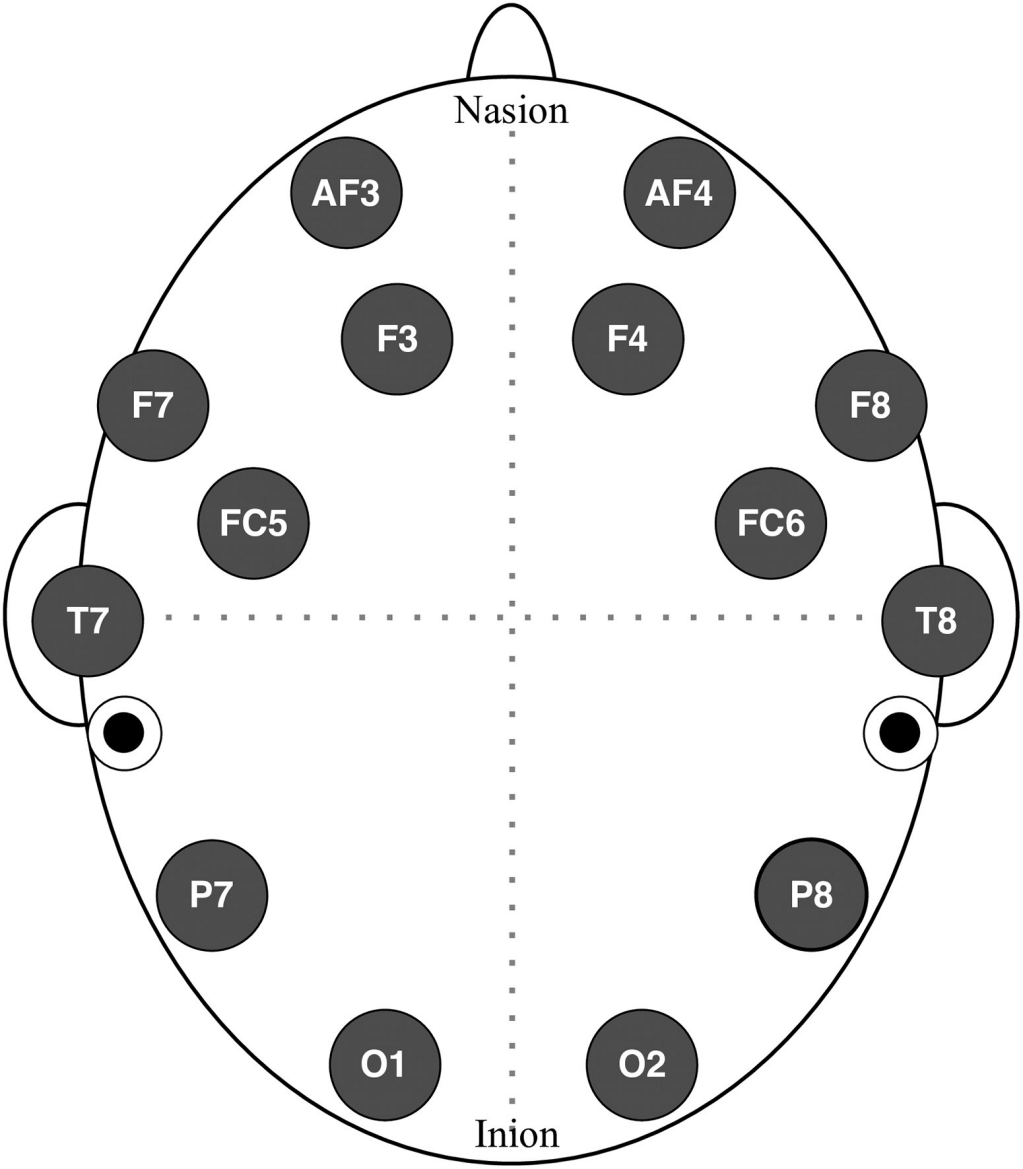
Electrode placement of the EMOTIV Epoc+ EEG headset (AF: Anterior-Frontal, F: Frontal, FC: Fronto-Central, T: Temporal, P: Parietal, O: Occipital), where odd numbers denote left hemisphere and even numbers represent right. Black dots indicate location of reference electrodes (“device ground” or “chassis ground”).

Subjects were instructed to stay still while capturing EEG readings, to reduce artifacts of head motion. The EMOTIV Epoc+ headset was used to collect signals in a calm and silent room. Wang et al. [22] had compared the EMOTIV headset with a clinical grade EEG device and confirmed that it produces reliable signals. Fig 1 shows the electrode distribution of the headset. The Epoc+ headset has 14 wet electrodes, and saline solution was used to increase their conductance. It took 10 to 15 minutes to set up the headset for each subject. A software package comes with an accessory to inject time-stamp markers, which can be used to separate trials and phases. The device was precisely tested before the experiment. The effect of ambient electromagnetic waves, including power lines, was minimized by isolating the experimental location, away from EMI (electromagnetic interference) of noise-producing equipment.

### 1.2 Trial organization

**Fig 2:**
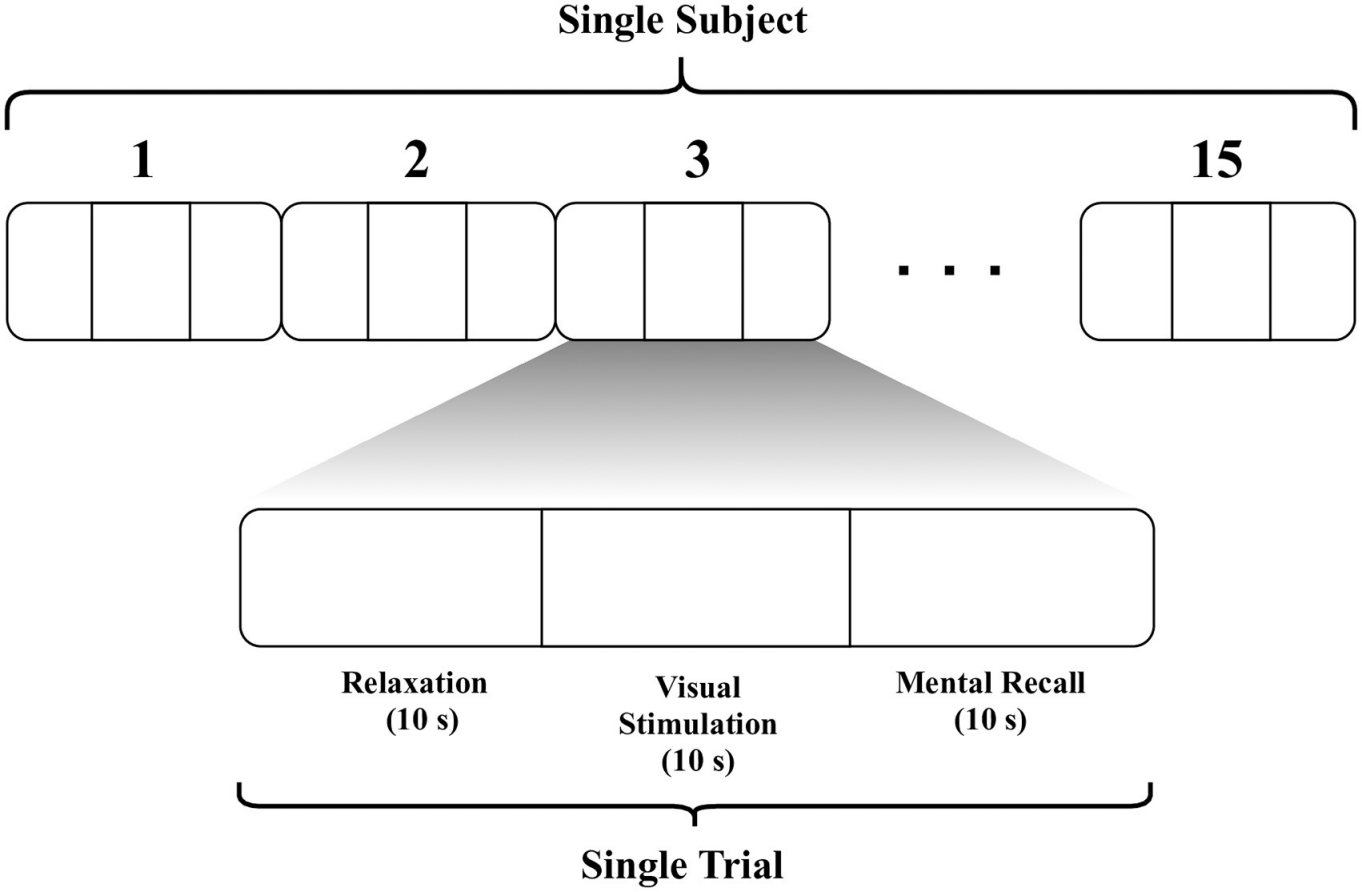
Structure of experiment: Each subject underwent 15 trials, and each trial comprised three phases. The stimulation phase necessarily precedes recall phase, and relaxation phase also serves as a refractory period, an implicit interval between trials, to be consistent with similar studies. (Each subject has tested for about 10 min).

Each experimental trial included three main phases—relaxation, visual stimulation, and mental recall—as shown in Fig 2. Calm music (“Magic Forest,” by Alexander Blu) was used to relax each subject with closed eyes for 10 s. Then a four-digit random number (white characters on black background) was shown on a screen (17” LCD monitor, 1366 × 768 pixel resolution) for 10 s. Finally, the subject was instructed to imagine the number seen on the screen for 10 s with closed eyes. The four-digit number was used assuming it helps to evoke more distinctive brain signal patterns in visual and mental recall phases. A single trial consisted of these three phases, and trials were recorded continuously with 128 Hz sampling rate. For each subject, 15 trials were conducted, separating trials using time-stamped markers. An inconvenience of the wet electrode system is that the electrodes dry out over time, lowering signal quality. Therefore, the time allocated to each subject was determined by the interval which allowed consistent signal quality.

### 1.3 Pre-processing and visualization

One way of visualizing collected EEG data is by plotting a topographic distribution. Even though topographs are usually used in geomatics, here the power distribution is plotted as the third dimension (instead of elevation) as a contour map. First, the data was normalized and converted to the frequency domain, using Discrete Fourier Transform (DFT) to calculate the power of each channel:

$$\begin{array}{c} \hat{x}\left[n\right]=\frac{x[n]-\text{min}(x)}{\text{max}(x)-\text{min}(x)}, \end{array}$$ (1)

where *x*[*n*] is a sequence of raw samples and $\hat{x}\left[n\right]$ is the corresponding vector of normalized samples. The DFT is calculated as

$$\begin{array}{c} \hat{X}\left[k\right]=\sum_{n=0}^{N-1}\hat{x}\left[n\right]e^{-j(\frac{2\pi }{N})nk}, \end{array}$$ (2)

where $\hat{X}\left[k\right]$ are the frequency-domain coefficients, *N* is the window size, and *k* = 0, 1, 2, ⋯, *N* − 1. Finally power is calculated as

$$\begin{array}{c} P=\hat{X}\cdot \bar{\hat{X}}=\sum |\hat{X}{\left[k\right]|}^{2}, \end{array}$$ (3)

where $\bar{\hat{X}}$ is the complex conjugate of the sample sequence, and *P* is power (strictly speaking, energy) of the channel. After calculating the power values, Natural Neighbor Interpolation [23] is used to infer power values between the electrodes. Plotting the contours for the calculated values gives presumed topographic fields. Our MATLAB code is shared on GitHub https://github.com/ijmax/EEG-processing/blob/master/topograph.m.

**Fig 3:**
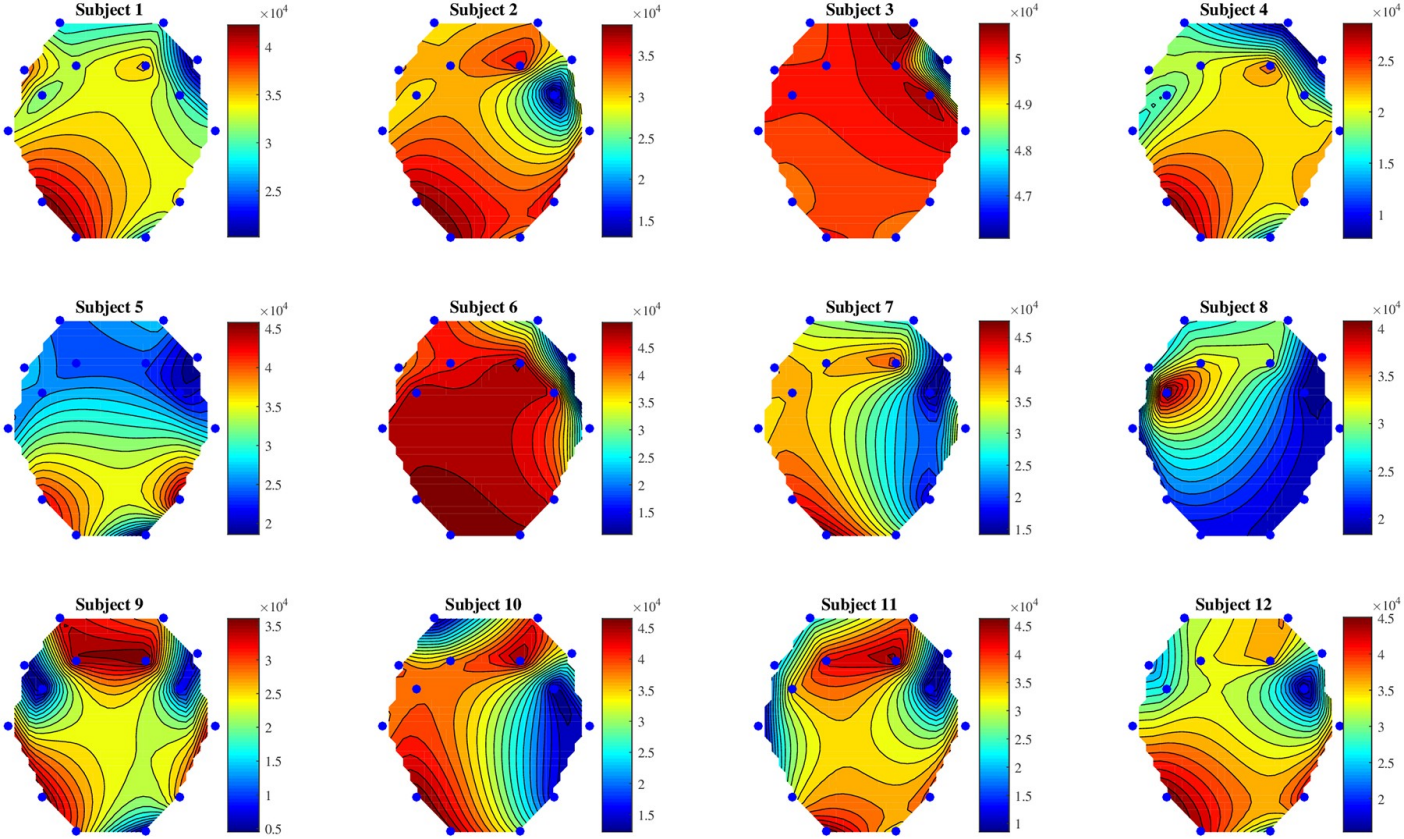
Topographic plots of 12 subjects for relaxation phase. (Maroon color represents high power values, and dark blue represents low).

The three trial phases (relaxation, visual stimulation, and mental recall) were separated from the continuous signals and saved as separate datasets. Each phase was set to fixed duration to maintain consistency, but the actual length differed slightly from phase to phase. Each phase for each trial for each subject had around 1280 samples because each signal was recorded at 128 Hz sampling rate for 10 seconds. The sample length of each phase was truncated to a fixed size (1280) by removing excess samples from the end. When using 14 electrode channels, each phase can be considered a 14 × 1280 matrix. Each phase set contains 180 trials, because 12 subjects were recruited and 15 trials were conducted for each subject. Fig 3 shows the topographic distribution plots of 12 subjects. Clear differences among these power distributions can be seen through this visualization. Every subject seems to have a uniquely personal brain wave patterns, even when performing the same task. Also, maximum and minimum power values differ from subject to subject.

### 1.4 Data augmentation

Data augmentation is a technique to increase the diversity of training data to improve results and avoid overfitting without collecting new data. Due to lack of trials per subject in our experiment, data augmentation techniques were used to expand the dataset. These techniques are usually domain-specific. Therefore, three signal augmentation techniques which have been used to improve classification performance of wearable sensor data was used [24].

**Fig 4:**
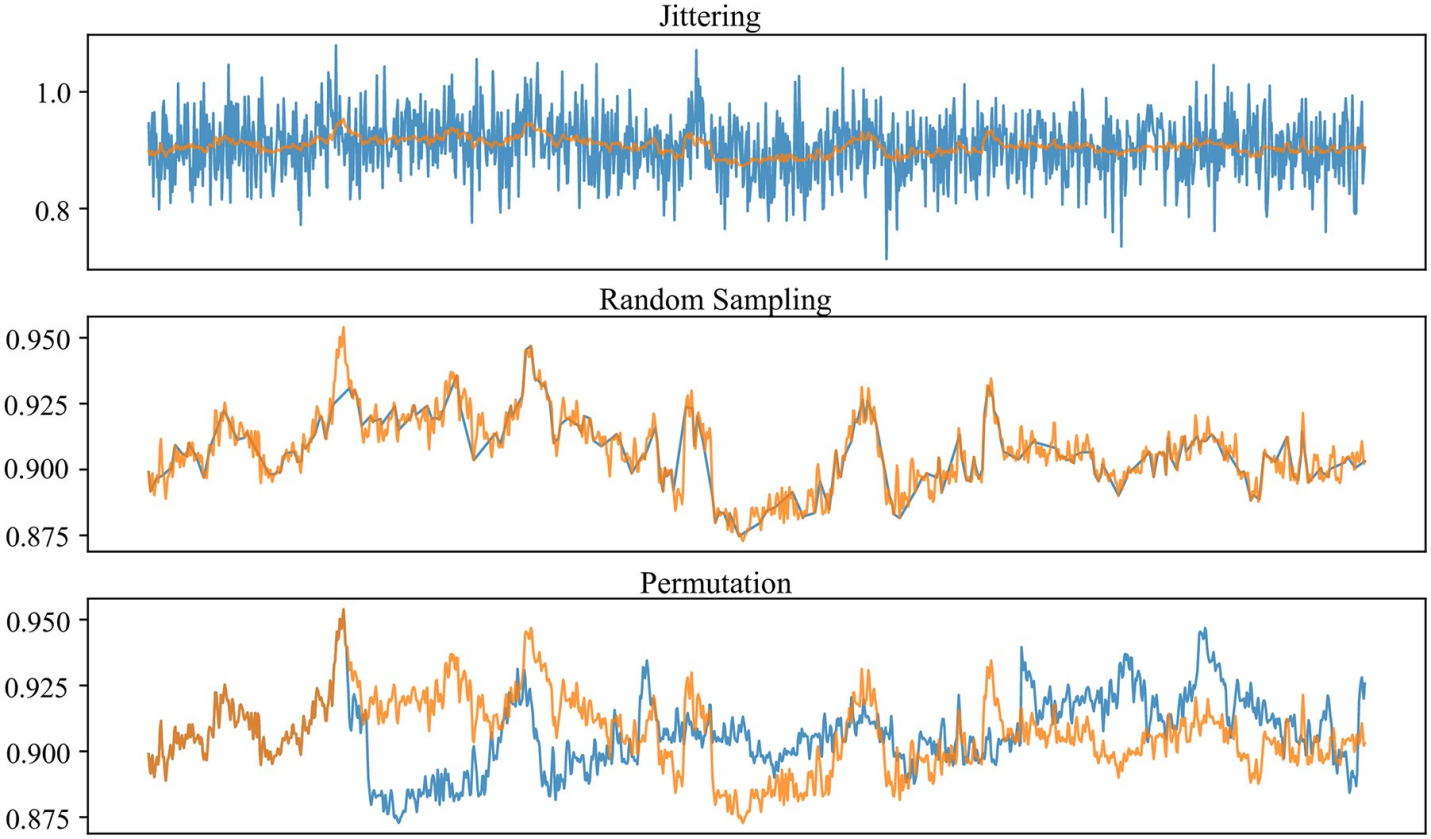
Visualization of raw and augmented signals subject to jittering, random sampling, and permutation techniques. Orange trace represents the original raw signal, and blue trace represents the augmented signal in each plot.

Fig 4 shows visualization of raw and augmented signals using following techniques:

- *Jittering*: This is a process of adding random noise to the signal. Normally distributed random noise was generated with standard deviation of 0.05.
- *Random sampling*: In this process, some samples are randomly removed, interpolation of remaining samples recovering the original length of the signal.
- *Permutation*: In this process, the temporal location of within-window events are randomly shuffled. 4 randomly selected segments with random length (minimum 5 samples) were altered randomly. To allow symmetry testing such as IHAR, time-wise alignment must be preserved, so the same permutation was applied to all 14 electrode channels.

Ten trials were separated after shuffling (to avoid bias from ordering such as learning effects) from each subject as training data and each 10 second trial was used to generate 10 instances from each augmentation technique, resulting 300 new trials. Final training set contained 12 × 310 = 3720 trials in total. Remaining 5 trials for each subject were kept as testing set. The same process was performed for all three phases.

**Fig 5:**
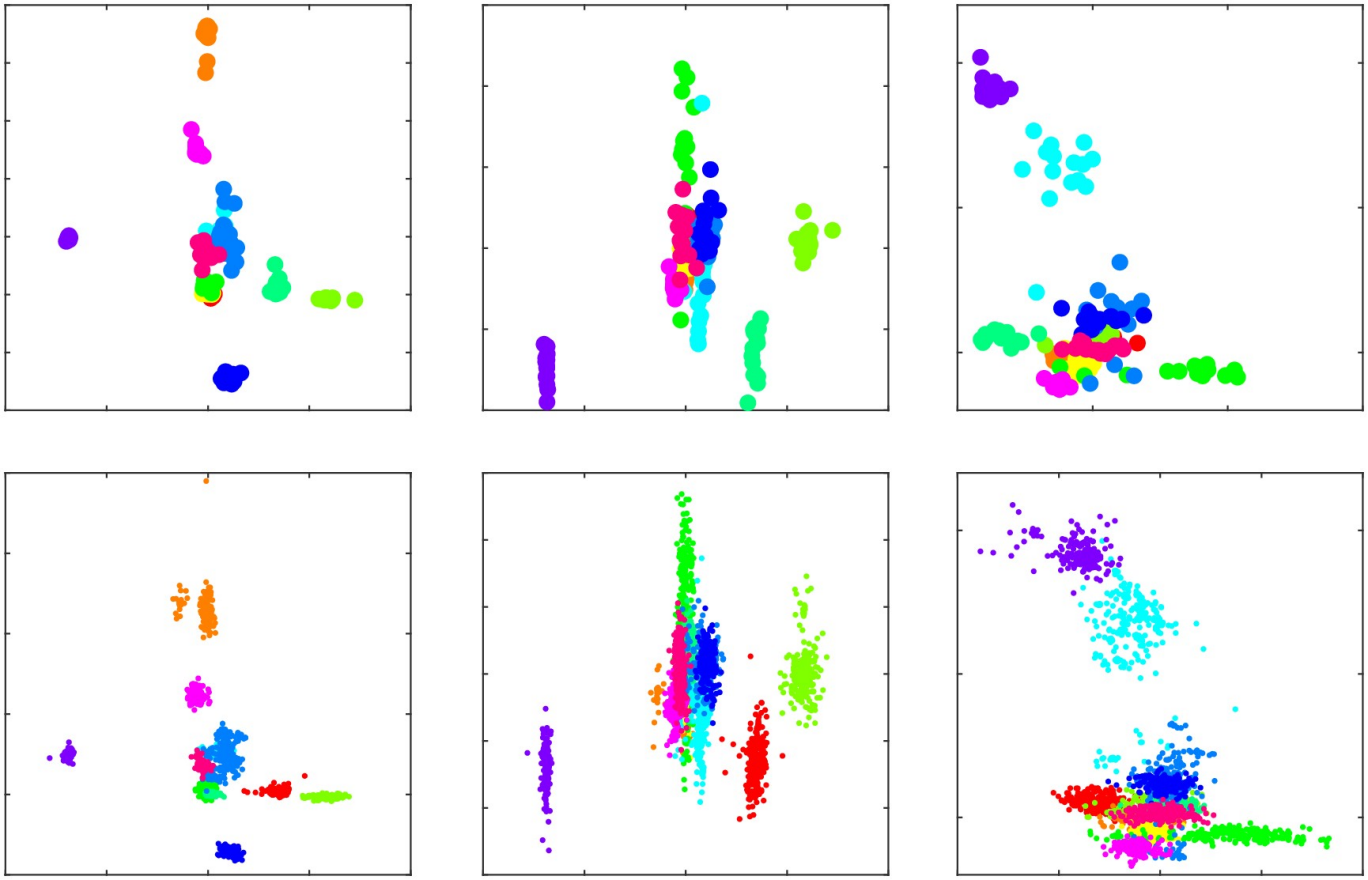
Distribution of features before and after the augmentation, using color to distinguish respective subjects. Top 3 plots show features of 10 training trials, while bottom 3 plots show corresponding features of augmented trials.

Scatter plots of some selected features as seen in Fig 5 were used to confirm that distribution of features of augmented signals were not changed significantly. According to the scatter plots, augmentation has widened the margins of each class while preserving the separable nature of features.

### 1.5 Inter-Hemispheric Amplitude Ratio (IHAR) vs. other features (LC, WL, SSC, AR)

There is asymmetry between brain hemispheres [25]. In human neuroanatomy, brain asymmetry can be categorized into two classes: (a) neuroanatomical differences such as neuronal densities, size of regions, etc., and (b) functional differences [26]. Such asymmetry can be seen in the power distributions of EEG data. All standard electrode placement systems (10-20, 10-10, 10-5) are bilaterally symmetric across the inion-nasion line, the median or sagittal plane. Also, most experimental results have shown that time-domain data contains discriminative aspects such as Autoregressive (AR) features [1], matching peaks [13, 27], and statistical features [28]. IHAR is also calculated in the time domain, and symmetric electrode pairs are needed. The proposed feature extraction method has two stages: (1) smoothing and (2) calculating ratio of corresponding channel pairs. Instead of using frequency-domain low-pass filtering for smoothing, a moving average (MA) filter [29] was used to remove high frequencies and random noise. The MA filter, applied in our analysis only for the IHAR feature, is an efficient way of extracting low-frequency signals compared to frequency-domain low-pass filters. It can also be calculated quickly, so is appropriate for modest embedded systems, such as anticipated practical deployment. Furthermore, power line noise can be easily removed from a captured raw signal [30].

**Fig 6:**
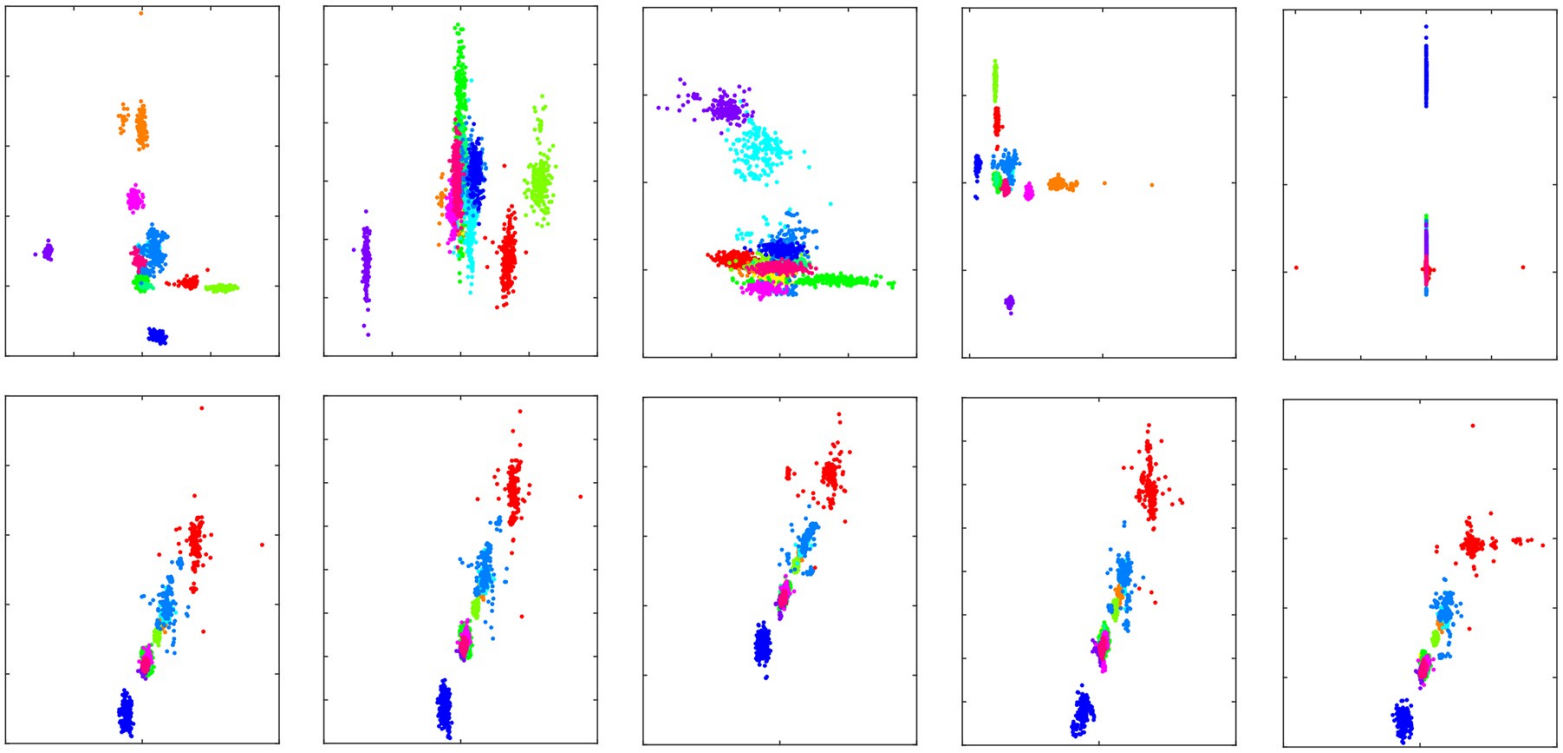
Comparison of selected feature distribution of IHAR and LC. Top 5 show scatter plots of IHAR, while bottom 5 plots show LC of corresponding feature pairs.

A similar derived parameter, dubbed “laterality coefficient” (LC), had been introduced [31] to study motor rehabilitation after a stroke. However, LC has not been used to find subjective differences among human subjects. Comparison of feature distribution of LC and IHAR can be seen in Fig 6. The same feature pair for both IHAR and LC were plotted, and IHAR showed more discrimination compared to LC as suggested by heterogeneous distributions across varied feature pairs.

**Fig 7:**
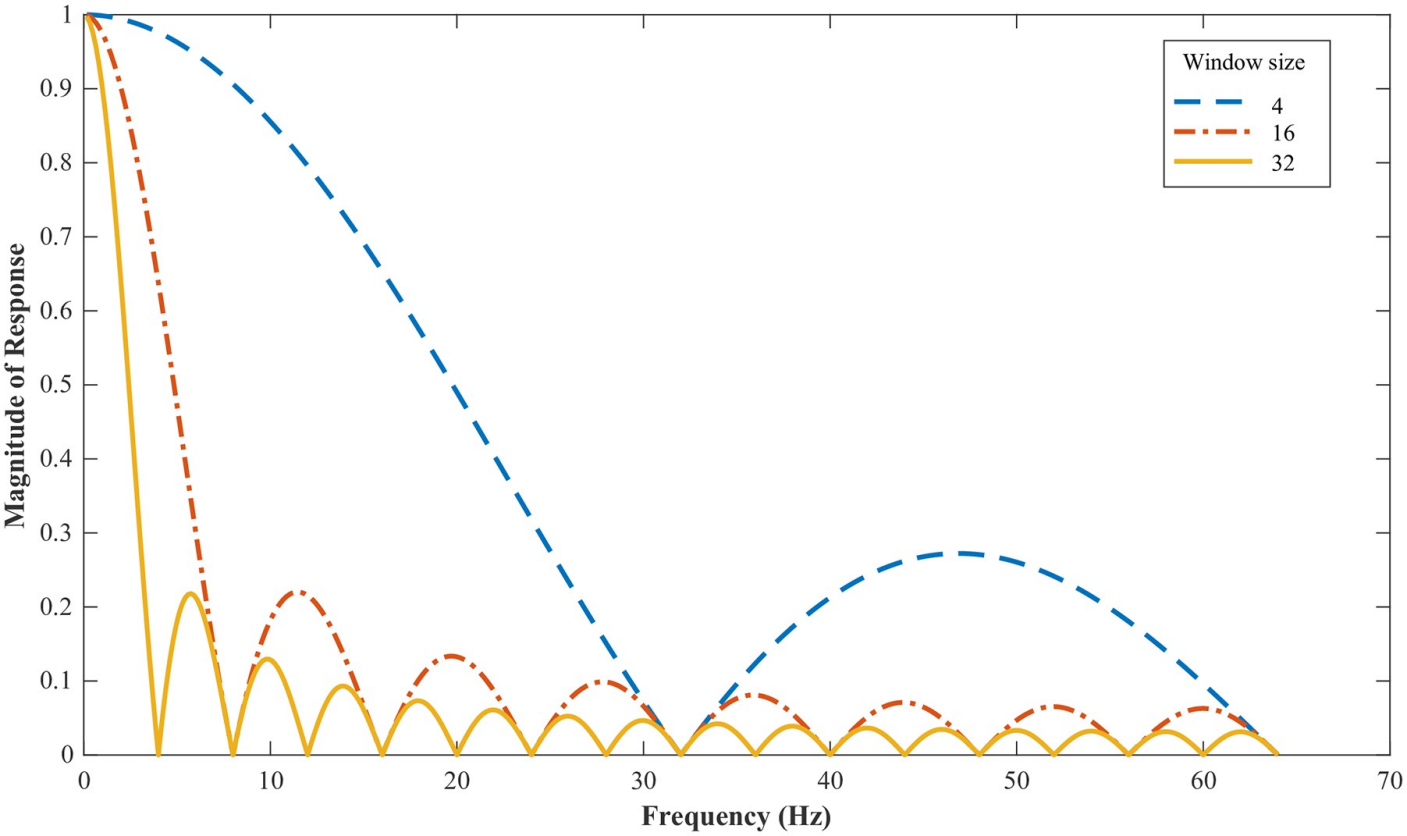
Frequency response of the MA filter with various window sizes.

Fig 7 shows the frequency response for a MA filter with three different window sizes. Three comprehensive analyses were performed, for window sizes 4, 16, and 32. Unlike other low-pass filters, MA filter does not have a sharp cut-off. The frequency response of an MA filter can be expressed as

$$\begin{array}{c} H\left(\omega \right)=\frac{1}{L}(\frac{1-e^{-j\omega L}}{1-e^{-j\omega }}), \end{array}$$ (4)

where angular frequency *ω* = 2*πf*, *e*^−*jω*^ is complex phasor of filter transfer function, and *L* is window size. As seen in Fig 7, when the window size is 4, the cut-off frequency (at which magnitude is attenuated by 3 dB so full-scale gain is $1/\sqrt{2}\approx .7$) is ∼15 Hz (*f*_*s*_ = 128 Hz), so the passband includes Delta (*δ*), Theta (*θ*), and Alpha (*α*) frequency ranges. Only *δ* band can be extracted when window size 16 or 32 is used. Most cognitive tasks, including relaxation and mental recall, produce low-frequency EEG signals [32]. Therefore, the above-mentioned window sizes were used to extract information in low-frequency signals. Furthermore, this filtering process eliminates power line noise, which “hum” lies between 50–60 Hz. The MA filter can be expressed as

$$\begin{array}{c} x_{A}\left[n\right]=\frac{1}{L}\sum_{k=0}^{L-1}x[n+k], \end{array}$$ (5)

where *L* is the window size, and *x*[⋅] are the time-domain samples. Finally, IHAR is calculated by dividing samples of a left channel by samples of its corresponding right channel:

$$\begin{array}{c} x_{\text{IHAR}}\left[i,\tilde{n}\right]=\frac{x[\text{left}[i],\tilde{n}]}{x[\text{right}[i],\tilde{n}]}, \end{array}$$ (6)

where *i* = 1, 2, …, 7 or total number of channel pairs, $\tilde{n}=1,2,\ldots ,$ total samples in the phase sequence. left[⋅] & right[⋅] are left and right channels lists arranged to align corresponding bilateral pairs, as in

$$\begin{array}{lllllllll} \text{left} & = & \text{AF}3, & \text{F}7, & \text{F}3, & \text{FC}5, & \text{T}7, & \text{P}7, & \text{O}1;\\ \text{right} & = & \text{AF}4, & \text{F}8, & \text{F}4, & \text{FC}6, & \text{T}8, & \text{P}8, & \text{O}2. \end{array}$$

For simplicity, henceforth the “IHAR” subscript is elided, and *x* is understood to refer to *x*_IHAR_.

To validate performance of the proposed feature, several time-domain features were calculated for comparison:

- *Laterality Coefficient (LC)*: ratio of difference and sum of a bilateral EEG pair, (*L* − *R*)/(*L* + *R*).
- *Waveform Length (WL)*: cumulative length of a particular segment of a signal.
- *Slope Sign Change (SSC)*: number of times the slope of a signal changes its sign.
- *Auto-regressive coefficients (AR)*: AR modeling is a process of predicting future values based on past values of time series data. Scalar values which model the prediction.

Except for LC, these features have especially been used in EMG analysis studies, including limb movement classification. Geethanjali et al. [33] conducted a performance comparison of some of these features using LDA classifier and obtained 67–100% accuracy range for pair-wise mental tasks classification. Each feature was extracted using a sliding window similar to MA filter process applied in our calculation of IHAR, so the extraction process was consistent for all features types. To calculate these four comparing features, some functions in “Myoelectric control development toolbox” developed by Chan et al. [34] were used. No smoothing, low-pass filtering, or averaging was applied to these compared features (besides IHAR).

When calculating each of these four features, the kernal window convolves through rows of the trial phase matrix (14 × 1280). The size of the output matrix after feature calculation depends upon the window size. Then the output matrix is flattened in row-major order to make a feature vector and fed to a classifier.

### 1.6 Classification

The extracted feature set was tested with several ML techniques to determine which algorithms show high accuracy for person identification. Unlike authentication, person identification is a straightforward classification problem, so traditional ML algorithms can be applied. Not knowing a priori which are best suited for this problem domain, we compared four well-known techniques.

#### 1.6.1 Linear Discriminant Analysis (LDA)

LDA [35, 36] is most commonly used as a dimensionality reduction method, similar to Principal Component Analysis (PCA). Assuming equal covariance for each class, and that conditional classes are multivariate, the LDA discriminant function *δ*_*k*_(*x*) (which separates inferred classes) can be defined as in Eq 7 and the classification rule shown in Eq 8:

$$\begin{array}{c} \delta_{k}\left(x\right)=\text{log}\pi_{k}+x^{T}\Sigma^{-1}\mu_{k}-\frac{1}{2}\mu_{k}^{T}\Sigma^{-1}\mu_{k}, \end{array}$$ (7)

where *π*_*k*_ denotes the prior probability that an observation belongs to the *k*th class, Σ is the common covariance matrix (not to be confused with the ∑ operator for summation), and *μ*_*k*_ is the mean of class *k*. The classification rule can be defined as

$$\begin{array}{c} \hat{G}\left(x\right)=\underset{k}{\text{arg}\;\text{max}}\left(\delta_{k}\left(x\right)\right). \end{array}$$ (8)

#### 1.6.2 Quadratic Discriminant Analysis (QDA)

The main difference of QDA from LDA is that QDA relaxes the assumption that the inputs of every class have the same covariance. Also, class decision boundaries are not linear but quadratic [37]. Eq 9 shows the QDA discriminant function. The classification rule is the same as that for LDA.

$$\begin{array}{c} \delta_{k}\left(x\right)=\text{log}\pi_{k}-\frac{1}{2}{\left(x-\mu_{k}\right)}^{T}\Sigma_{k}^{-1}\left(x-\mu_{k}\right)-\frac{1}{2}\text{log}\left|\Sigma_{k}\right|, \end{array}$$ (9)

where Σ_*k*_ is the covariance matrix of the *k*th class.

#### 1.6.3 Support Vector Machine (SVM)

SVM, a.k.a. Support Vector Network, is also a classification technique, finding the hyperplane which maximizes the margin between two classes [38]. So-called support vectors define the hyperplane that makes the separation. Linear, polynomial, Radial Basis Function (RBF), and sigmoid are the basic types of SVM kernels [39]. The SVM decision function can be expressed as

$$\begin{array}{c} f\left(x\right)=\text{sign}(\sum_{i=1}^{N}\alpha_{i}y_{i}K\left(x_{i},x\right)+b), \end{array}$$ (10)

where *N* is the size of training data, *K* is the kernel function that measures similarity between *x*_*i*_ (support vector) and *x* (feature values), *α*_*i*_ is Lagrange multiplier, *y*_*i*_ represents the membership class of each datum (±1), and *b* is a numeric constant. We used linear, polynomial, and RBF kernels to determine accuracy of the extracted features, respectively described following.

Linear SVM: a linear kernel was used, and its kernel function can be expressed as inner (“dot”) product of support vector and feature values:

$$\begin{array}{c} K\left(x_{i},x\right)=x_{i}^{T}\cdot x. \end{array}$$ (11)

Quadratic and cubic (*d* = 2 and 3) SVM polynomial kernels were used,

$$\begin{array}{c} K\left(x_{i},x\right)={\left(x_{i}^{T}x+c\right)}^{d}, \end{array}$$ (12)

with *c* = 1 for both cases.

Medium and Coarse Gaussian SVM: An RBF kernel function was used with *σ* = 16 and 64:

$$\begin{array}{c} K\left(x_{i},x\right)=\text{exp}(-\frac{{∥x_{i}-x∥}^{2}}{2\sigma^{2}}), \end{array}$$ (13)

where ∥*x*_*i*_ − *x*∥ is Euclidean distance, and standard deviation *σ* determines the width of the Gaussian kernel.

#### 1.6.4 k-Nearest Neighbor (kNN)

kNN is the simplest machine learning algorithm, measuring distance to a given data point. *k* stands for the number of neighbors which should be taken into account, and accuracy can be varied by adjusting its value. There are several distance measurements, including Euclidean distance (Eq 14):

$$\begin{array}{c} d\left(x,x^{\prime }\right)=∥x-x^{\prime }∥=\sqrt{\sum_{i=1}^{k}w_{i}{\left(x_{i}-x_{i}^{\prime }\right)}^{2}}, \end{array}$$ (14)

where *d*(*x*, *x*′) is the distance between points *x* and *x*′, and *w*_*i*_ is edge weight.

kNN (*k* = 1) with Euclidean distance with equal weights were used in this analysis.

After extracting the IHAR feature, accuracy was tested in several ways. First we tested three phases with all 14 electrodes (7 electrode pairs). Further, accuracy was checked for three trial phases while reducing the number of electrodes. An augmented training dataset (3720 trials) was used to train all the above-discussed ML models, and remaining 60 trials (5 trials per each subject) with original signal data was used to test the models. K-fold cross-validation with 10 folds was used to validate the trained model. Accuracy was calculated as the ratio of correctly classified trials to all testing trials.

## 2 Results and discussion

After separating three trial phases from each single EEG signal, each phase was visualized using a custom MATLAB script. Fig 3 depicts the distribution of power of the relaxation phase, over crania of 12 subjects, showing some differences after channel-wise normalization (linear scaling with bias to remap to 0–1). Original unipolar (unbalanced) unsigned electrode data remain so after normalization. Clear asymmetry can be observed across all the nasion-inion lines. The EMOTIV Epoc+ headset uses mastoid bones as reference electrodes which helps to maintain a fair alignment of electrodes. Moreover, symmetry of electrodes was roughly checked by measuring distances from the sagital line before recording. However, phrenology discredited measurement of head shapes, and the helmet, cap, or headset were sometimes not perfectly aligned with the subject’s sagital plane. Perfectly consistent electrode alignment is practically impossible (without extreme invasive techniques such as Neural Lace [40]).

Data augmentation is used in machine learning when the training dataset is significantly small. Even if large number of trials provide more reliable classification results, such collection reduces the practicality in this domain. Therefore, data augmentation provides extra convenience to users when they record their brain signals for training. In this experiment, augmentation techniques and parameters were selected heuristically. However, drastic changes of parameters could affect classification results.

To check whether the extracted features are specific to individual subjects, statistical hypothesis testing was performed. Expressing “extracted IHAR features do not have subjective discrimination” as the null hypothesis, MANOVA (Multivariate Analysis of Variance) was used for this test. The null hypothesis was rejected for all relaxation, visual, and mental recall phases with the *p*-values 0.0043, 0.031, and 0.0037, respectively, which are all less than conventional threshold 0.05.

**Fig 8:**
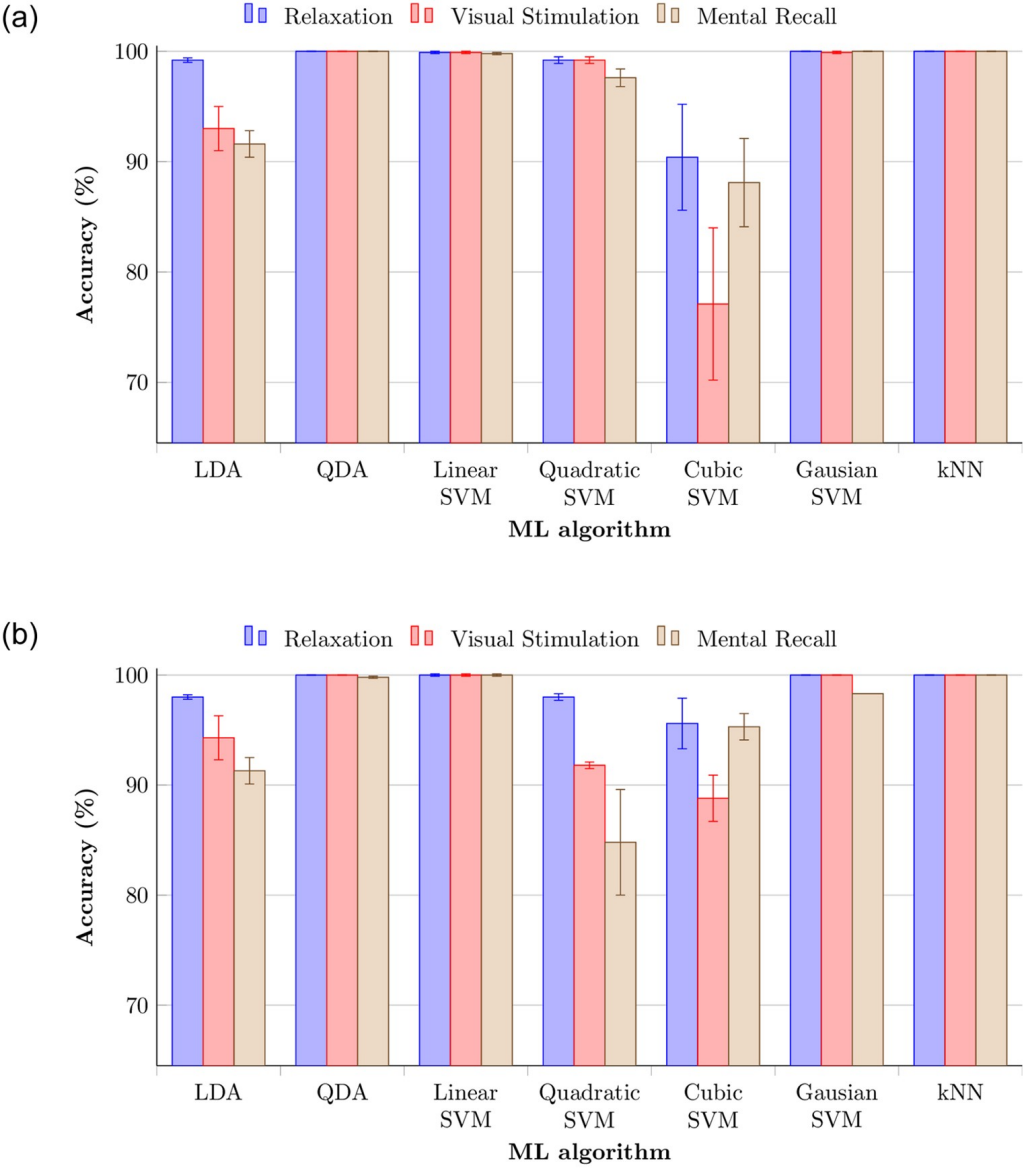
Accuracy of three phases with 14 electrodes for various discriminant analysis, support vector machine, and k-nearest neighbor techniques with OVO method. (a) Validation accuracies. (b) Testing accuracies.

**Fig 9:**
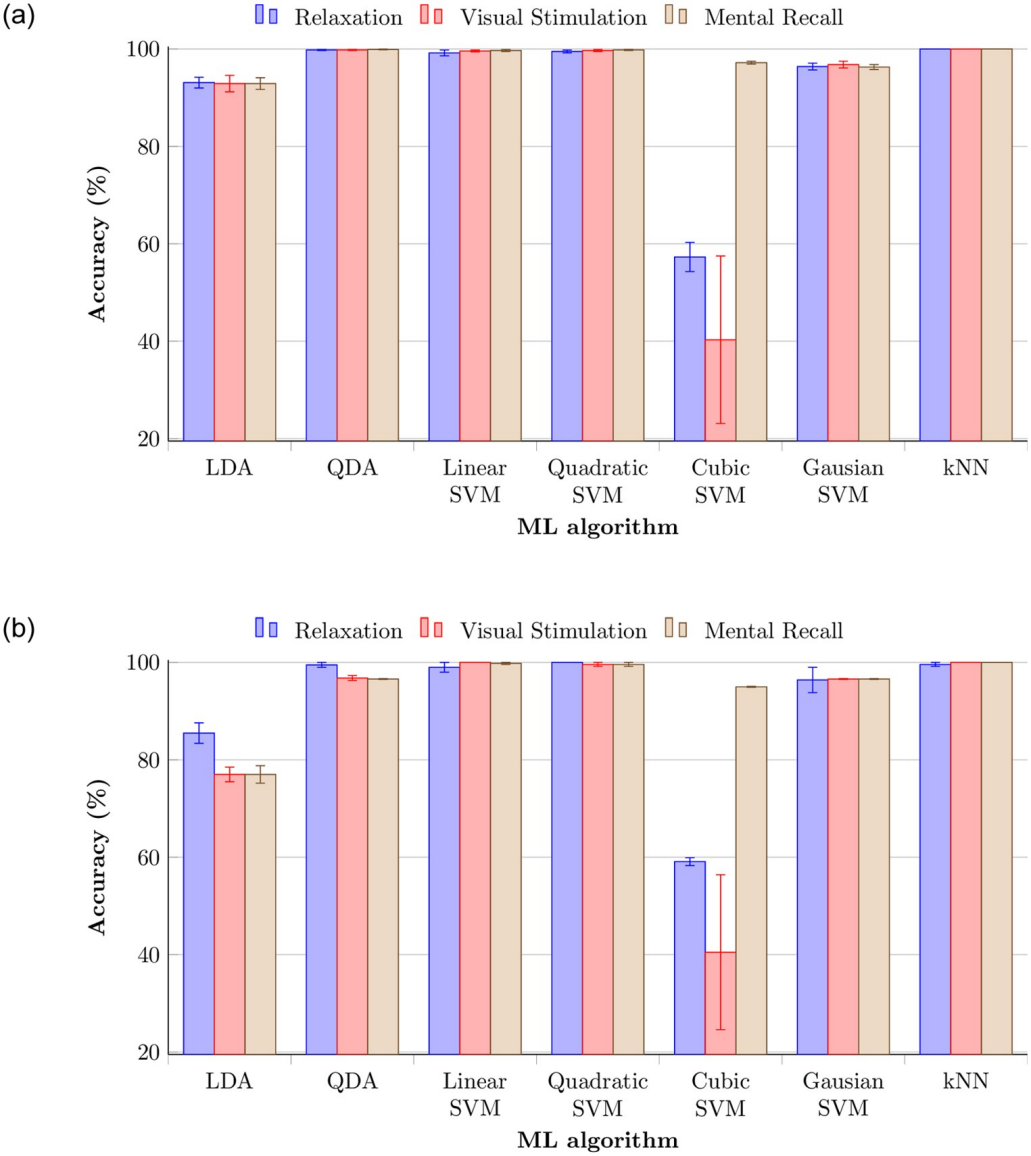
Accuracy of three phases with 14 electrodes with OVA method. (a) Validation accuracies. (b) Testing accuracies.

Validation and testing accuracies of the three phases with 14 electrodes for the above-discussed machine learning techniques are shown in Fig 8. The data of each subject was trained against that of every other subject, which creates *N*(*N* − 1)/2 = 66 classifiers, the so-called One-vs.-One (OVO) multiclass classification method. This method shows even better accuracy than One-vs.-All (OVA), albeit only slightly. Validation and testing accuracies for the same data set with OVA method can be seen in Fig 9. OVO is usually better than OVA because class imbalance occurs using OVA method even if OVA is faster in training. QDA, linear SVM, Gaussian SVM, and kNN showed the highest validation accuracy for all three phases. The testing results also were close to 100% for the same ML techniques. Furthermore, relaxation phase showed higher validation and testing accuracy than other phases. Signal patterns evoked in the relaxation phase seem more stable compared to those from the other phases. Lateral asymmetry or energy difference of alpha activity between two hemispheres during the relaxation explains the high performance of relaxation phase [41]. Quadratic SVM showed better validation accuracy, but testing accuracy was slightly lower. Even though cubic SVM acheived lowest validation accuracy among all techniques, testing accuracy was lightly increased. The relaxation phase generally yielded highest accuracy with OVO method.

**Fig 10:**
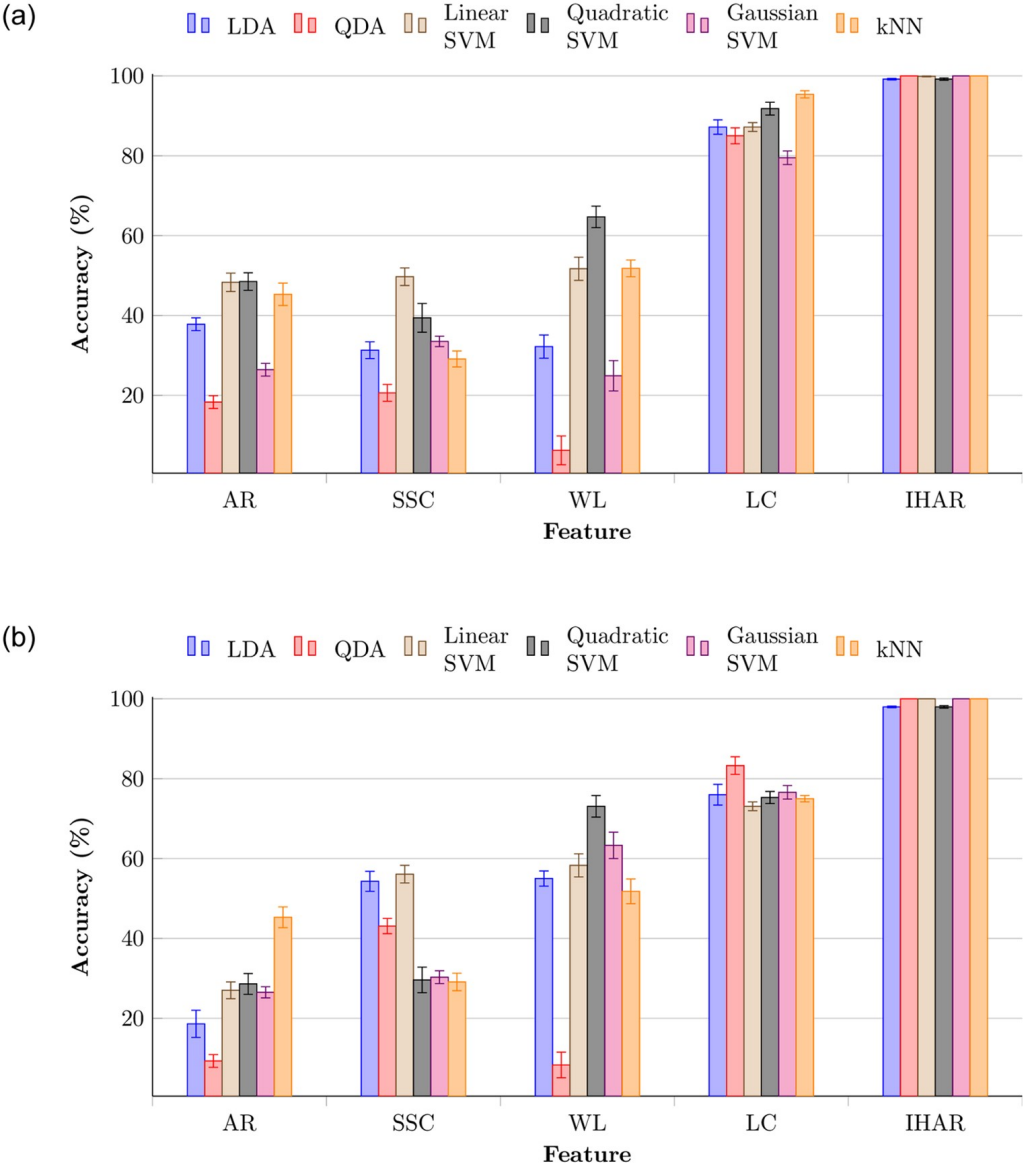
Comparison of accuracy across features (AR: Auto-regressive coefficients, SSC: Slope sign change, WL: Waveform length, LC: Laterality coefficient, and IHAR: Inter-hemispheric amplitude ratio) extracted during relaxation phase using all 14 electrodes. (a) Validation accuracies. (b) Testing accuracies.

Comparison of performances with other time-domain features for same conditions is shown in Fig 10. All 14 channels were used to test each feature extraction method. While IHAR achieved highest accuracy, LC was the next best-performing feature, achieving 95.4±0.9% validation accuracy for kNN classifier, and 83.3±0.1% maximum testing accuracy for QDA. The conceptual similarity of the LC feature to proposed IHAR explains why it achieved comparable results, especially for validation (but not testing). AR, SSC, and WL showed less than 50% validation accuracies for most of the ML techniques. Even though the AR feature has been used in many EEG-related studies, it did not show considerable results for any classifier.

After selecting the best ML algorithms among tested candidates, accuracy was checked for each electrode pair. Results can be seen in Table 1. The highest testing accuracy, 94.7±1.1%, was from the FC5-FC6 electrode pair for QDA with relaxation phase. According to the results, FC5-FC6 electrode pair yielded over 90% accuracy for most of the ML algorithms. Other frontal electrode pairs F7-F8 and AF3-AF4 were the next highest. Occipital electrodes (O1-O2) showed the worst accuracy, 43.5±2.8% for the linear SVM with relaxation task. In addition, O1-O2 showed the worst testing accuracy even for the visual stimulation phase in contrast to most research findings [42, 43]. In most of the studies, so-called oddball stimulus has been used to get the P300 (positive peak at 300 ms after the stimulation) spike from the occipital area. In our study, the image (four-digit number) was shown without making any changes (blinking or switching with another image) as visual stimulation. This explains the poor performance of occipital electrodes for visual stimulation. Several studies have considered frontal asymmetry in cognitive processes of the brain [44, 45]. Our results also showed considerable accuracy for the frontal electrodes for relaxation and mental recall phases.

**Table 1:** Validation and testing accuracies of each electrode pair for three task phases for various analysis techniques using IHAR.

ML algorithm	AF3-AF4		F7-F8		F3-F4		FC5-FC6		T7-T8		P7-P8		O1-O2	
	VA	TA	VA	TA	VA	TA	VA	TA	VA	TA	VA	TA	VA	TA
LDA	86.7 ± 1.9	70.5 ± 2.2	91.8 ± 1.4	83.5 ± 1.4	67.6 ± 1.5	50.1 ± 2.4	94.3 ± 1.4	87.4 ± 1.2	82.0 ± 1.6	69.1 ± 2.2	80.2 ± 1.3	65.0 ± 1.5	65.2 ± 2.2	47.1 ± 2.4
	85.0 ± 1.4	76.1 ± 1.3	91.2 ± 1.3	85.3 ± 1.8	62.9 ± 1.8	50.8 ± 2.5	90.2 ± 1.5	78.0 ± 1.7	77.2 ± 2.3	55.0 ± 2.2	79.3 ± 2.2	75.8 ± 3.2	62.3 ± 2.4	51.1 ± 1.7
	86.9 ± 1.6	73.6 ± 1.8	92.1 ± 1.1	74.6 ± 1.7	67.3 ± 1.6	60.0 ± 1.3	93.4 ± 0.8	89.1 ± 0.8	78.9 ± 1.9	59.8 ± 1.4	79.8 ± 1.9	77.8 ± 2.0	68.6 ± 2.6	59.3 ± 2.2
QDA	86.0 ± 1.2	73.3 ± 1.5	94.9 ± 1.0	88.3 ± 2.1	60.2 ± 2.7	57.1 ± 2.3	91.8 ± 0.8	**94.7 ± 1.1**	79.0 ± 1.6	80.6 ± 2.1	78.8 ± 1.7	73.3 ± 2.6	59.8 ± 1.3	55.3 ± 3.2
	81.4 ± 2.1	**90.1 ± 0.5**	86.1 ± 1.7	83.5 ± 1.2	50.8 ± 3.1	62.8 ± 0.8	88.5 ± 1.7	82.3 ± 0.8	75.8 ± 1.5	76.0 ± 0.8	75.4 ± 1.7	72.5 ± 1.1	55.8 ± 3.1	51.0 ± 1.4
	85.1 ± 1.8	84.0 ± 1.4	94.3 ± 1.3	81.5 ± 0.5	55.0 ± 2.7	55.3 ± 2.1	94.9 ± 1.1	**92.0 ± 0.1**	76.5 ± 2.0	68.5 ± 0.5	78.1 ± 1.7	76.8 ± 0.9	63.1 ± 1.7	59.3 ± 0.8
Linear SVM	89.1 ± 1.4	74.6 ± 1.8	93.1 ± 1.1	89.1 ± 2.3	70.2 ± 2.3	47.8 ± 2.3	95.1 ± 1.1	86.3 ± 2.2	84.0 ± 1.1	68.8 ± 2.6	83.1 ± 2.3	61.3 ± 2.3	70.6 ± 2.5	43.5 ± 2.8
	86.5 ± 1.2	73.6 ± 1.5	92.7 ± 1.2	83.1 ± 1.6	65.8 ± 2.1	52.5 ± 2.5	90.9 ± 0.5	79.3 ± 1.6	80.6 ± 1.1	57.1 ± 1.7	82.0 ± 1.8	73.3 ± 2.6	64.6 ± 2.1	47.5 ± 2.7
	88.7 ± 1.3	75.0 ± 2.3	93.8 ± 1.4	82.8 ± 1.9	69.0 ± 3.1	61.3 ± 1.3	94.1 ± 1.0	**90.0 ± 0.1**	80.6 ± 1.3	61.6 ± 1.3	81.6 ± 2.1	76.1 ± 2.3	70.9 ± 2.4	57.3 ± 1.7
Quadratic SVM	93.1 ± 1.3	80.5 ± 1.2	95.3 ± 1.5	85.3 ± 2.6	79.2 ± 1.5	57.0 ± 2.3	96.6 ± 0.1	83.1 ± 1.1	89.1 ± 0.1	77.3 ± 2.1	89.7 ± 1.2	74.1 ± 1.7	81.9 ± 2.3	60.0 ± 2.2
	89.1 ± 1.4	76.1 ± 1.5	96.0 ± 1.1	85.8 ± 0.8	76.3 ± 1.1	62.5 ± 1.4	95.3 ± 0.7	84.5 ± 1.1	87.4 ± 1.2	68.3 ± 1.9	86.8 ± 1.5	74.8 ± 2.2	76.8 ± 2.2	65.8 ± 3.4
	92.7 ± 1.1	82.5 ± 2.5	96.5 ± 0.6	78.5 ± 1.2	78.5 ± 2.5	67.1 ± 1.1	96.9 ± 0.8	**94.3 ± 0.8**	88.1 ± 1.6	73.3 ± 3.0	87.6 ± 1.9	74.8 ± 2.7	78.2 ± 2.8	53.3 ± 2.4
Gaussian SVM	79.1 ± 1.6	69.1 ± 1.2	86.9 ± 1.9	82.8 ± 2.6	54.0 ± 2.9	56.7 ± 2.7	90.1 ± 3.5	87.2 ± 2.2	76.1 ± 1.6	77.4 ± 2.8	73.0 ± 1.4	73.1 ± 2.6	53.4 ± 1.6	71.0 ± 1.4
	78.6 ± 1.3	77.6 ± 0.8	84.3 ± 1.5	83.3 ± 0.1	46.3 ± 1.7	49.1 ± 1.1	81.2 ± 1.2	82.8 ± 1.1	66.4 ± 1.8	53.5 ± 0.9	68.7 ± 1.8	68.3 ± 0.1	49.1 ± 1.9	45.8 ± 1.4
	76.9 ± 0.9	83.1 ± 0.5	91.6 ± 1.3	85.0 ± 0.1	48.4 ± 2.2	50.3 ± 1.0	90.5 ± 1.6	**93.3 ± 0.1**	71.2 ± 0.8	52.3 ± 1.1	73.1 ± 1.5	63.3 ± 1.1	54.1 ± 1.9	47.6 ± 1.1
kNN	95.6 ± 1.2	75.0 ± 1.2	98.1 ± 0.5	88.0 ± 2.1	86.1 ± 2.1	51.1 ± 3.6	97.2 ± 0.9	85.6 ± 1.3	93.7 ± 1.1	72.0 ± 1.9	93.6 ± 0.4	67.9 ± 3.1	89.4 ± 1.2	59.1 ± 2.7
	93.5 ± 0.8	82.3 ± 1.1	97.6 ± 0.5	88.3 ± 0.7	83.9 ± 2.4	58.1 ± 3.0	96.7 ± 0.9	83.5 ± 0.5	92.2 ± 1.4	75.0 ± 2.3	93.8 ± 1.5	72.5 ± 1.1	85.0 ± 1.3	49.6 ± 2.3
	94.1 ± 0.5	87.8 ± 1.3	98.1 ± 0.5	88.1 ± 0.9	85.8 ± 1.8	62.8 ± 1.7	97.2 ± 0.4	**94.6 ± 0.5**	91.4 ± 0.8	65.5 ± 1.7	93.4 ± 1.4	65.5 ± 1.7	86.6 ± 1.3	60.5 ± 2.6

VA: validation accuracy, TA: training accuracy

White (top row in each cell): relaxation; light gray (middle cellular row): visual task; dark gray (bottom row): mental recall

Testing accuracies ≥ 90% are emboldened.

**Fig 11:**
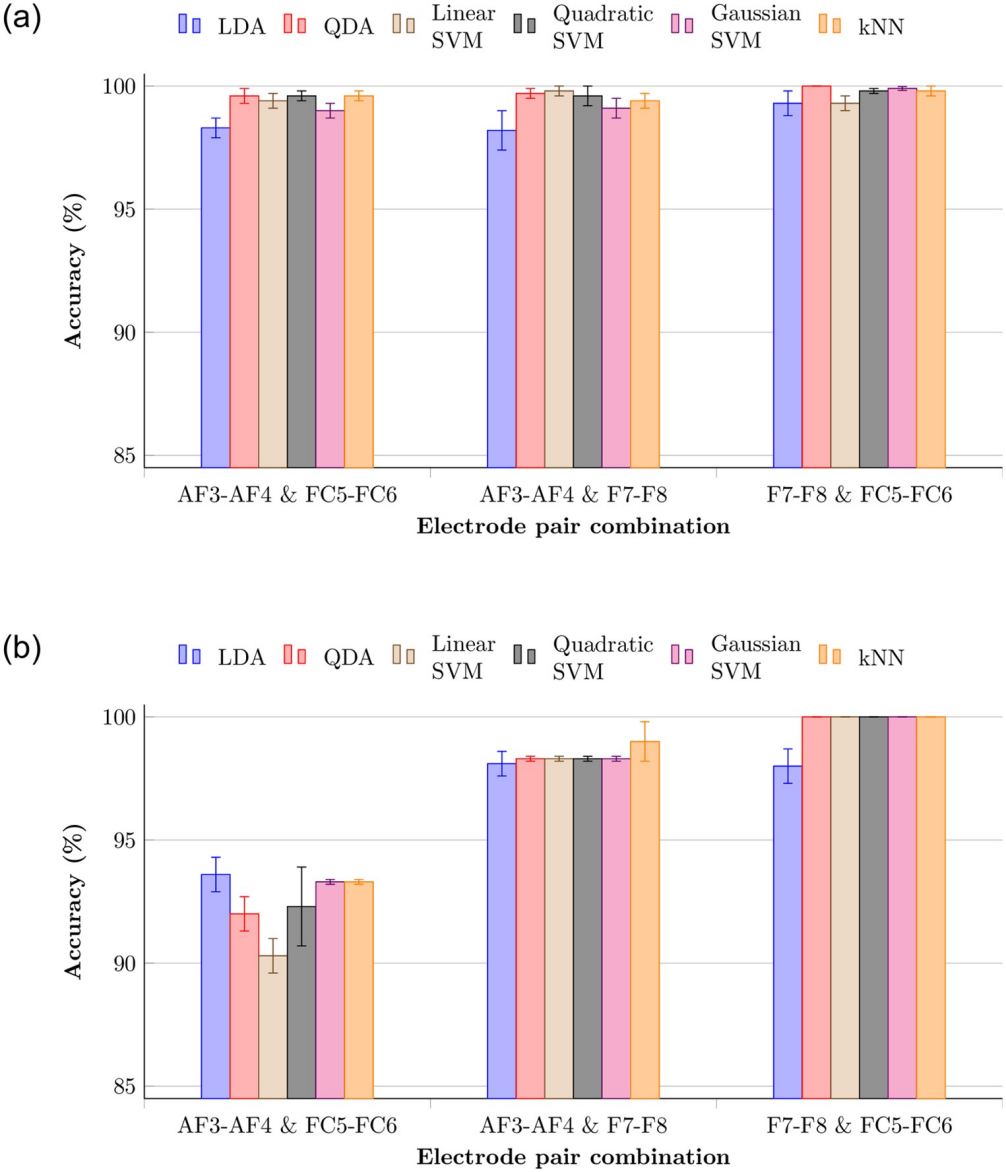
Accuracies of combination of 2 frontal electrode pairs which yielded higher accuracies. (a) Validation accuracies. (b) Testing accuracies.

In order to increase accuracy further with minimum electrode setup, the model was trained again by considering sets of electrode pairs. 100% testing accuracy was shown by the four frontal electrodes (F7-F8 and FC5-FC6) for all classifiers except LDA. As seen in Fig 11, accuracy increases when combining other frontal electrode pairs. A Receiver Operating Characteristic (ROC) curve, created by plotting the true positive rate (TPR) against the false positive rate (FPR), was plotted for all subjects with F7-F8 and FC5-FC6 electrode combination. The AUC (area under the ROC curve) value of all classifiers was unity, which means the trained model was a perfect classifier. Considering user-friendliness of specific hardware design for EEG biometrics, the anterior frontal electrodes AF3-AF4 pair is better than FC5-FC6, which is nearer to the coronal center. Therefore, the AF3-AF4 and F7-F8 electrode setup also was tested, and 99.0±0.8% (kNN), 98.3±0.1% (QDA), 98.3±0.1% (linear SVM), 98.3±0.1% (quadratic SVM), and 98.1±0.5% (LDA) testing accuracies were achieved for relaxation phase. The average AUC value for these cases lies between 0.98 and 1. These results seem to accommodate lighter devices which require less calibration time, such as Muse [46]. Even though entire hemispheres manifest asymmetry, the “frontal asymmetry” feature has been introduced in EEG analysis, calculated using frontal channels. Specifically, frontal asymmetry has been used in emotion and attention-related analysis [47, 48]. Since the frontal lobe is responsible for executive tasks [49], IHAR-based analysis for relaxation and mental recall phases shows high accuracy in frontal electrodes.

Specificity (true positive rate) and sensitivity (true negative rate) were calculated for electrode combinations which showed higher accuracies. Specificity is the ratio of number of trials that correctly classified as negatives to number of all negative testing trials, while sensitivity is the ratio of number of trials that correctly classified as positives to number of all positive testing trials. Models with high specificity values are better in the authentication domain because they decrease risk of granting access to imposters. As shown in Table 2, all electrode combinations achieved highest specificity value, which confirms suitability of IHAR method for this problem (better to tolerate occasional false rejection rather that allow false accepts).

**Table 2:** Comparison of performances for different number of electrodes.

ML algorithm	FC5-FC6			AF3-AF4, FC5-FC6			AF3-AF4, F7-F8			F7-F8, FC5-FC6		
	ACC	SP	SE	ACC	SP	SE	ACC	SP	SE	ACC	SP	SE
LDA	87.4	100	78	93.3	100	98	98.1	100	98	98.1	100	98
QDA	94.7	100	98	92.0	100	96	98.3	100	100	100	100	100
Linear SVM	86.3	100	76	90.8	100	95	98.3	100	100	100	100	100
Quadratic SVM	83.1	100	96	91.5	100	98	98.3	100	99	100	100	100
Gaussian SVM	87.2	100	80	93.3	100	97	98.3	100	98	100	100	100
KNN	85.6	100	84	93.3	100	100	99.3	100	100	100	100	100

ACC: Accuracy, SP: Specificity, and SE: Sensitivity

AF: Anterior Frontal, F: Frontal, FC: Fronto-Central

**Fig 12:**
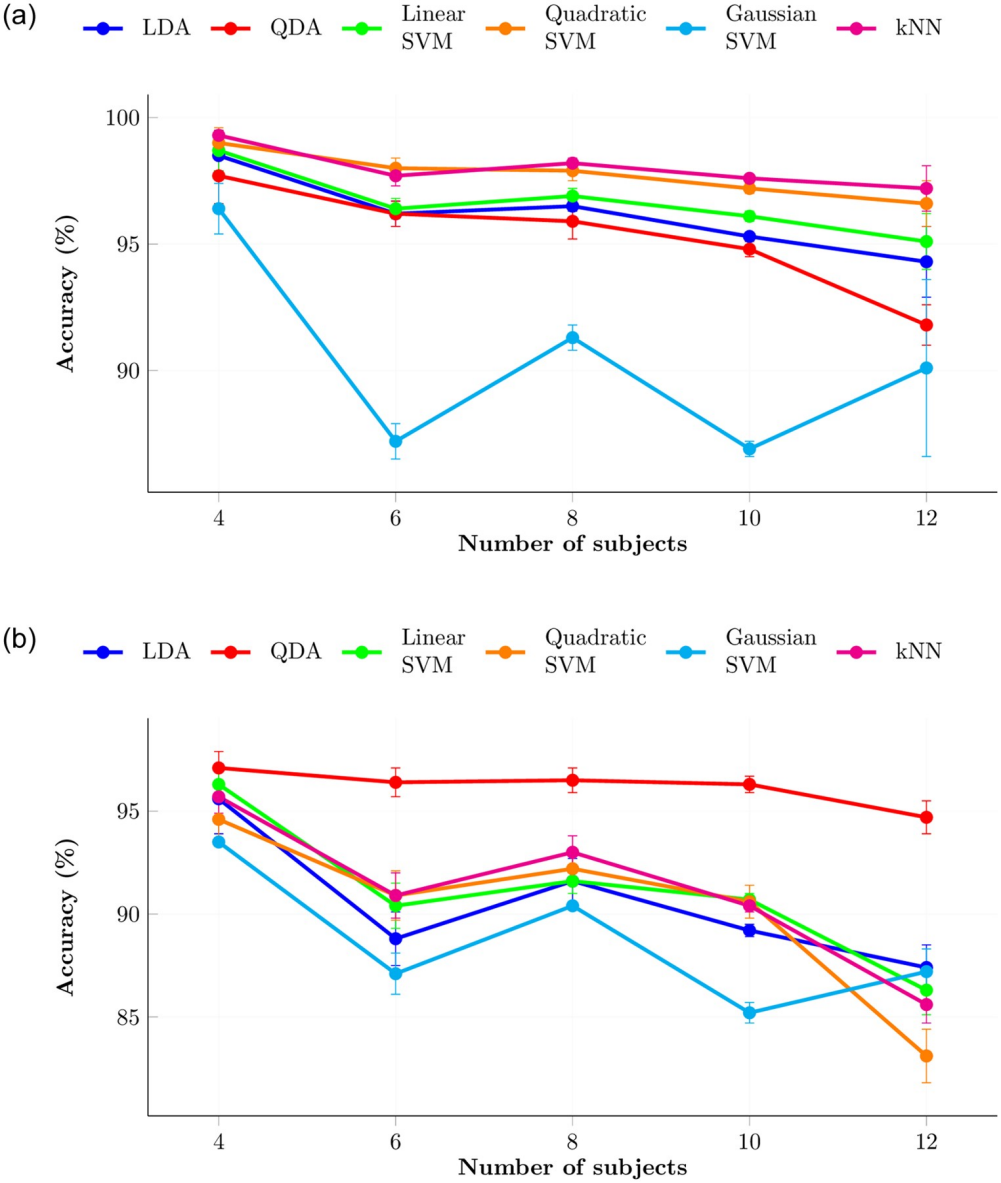
Variation of validation and testing accuracy for FC5-FC6 electrode pair of relaxation phase when increasing number of subjects using IHAR. (a) Validation accuracies. (b) Testing accuracies.

More information can be captured using high-resolution EEG devices. Compared with consumer-grade devices, clinical grade devices have more electrodes. Even though high accuracy can be achieved with the help of many electrodes, lighter electrode setup provides more user-friendliness. As shown in Table 3, other approaches achieved high accuracy using more channels, for compound or complex tasks. Our approach achieved the same accuracy with only four frontal electrodes for a simple task. However, accuracy of the models cannot be guaranteed when increasing the number of subjects. Fig 12 shows the general degradation of accuracy while increasing the number of subjects. Average accuracy was calculated for 10 random combinations of subjects with FC5-FC6 electrode pair, which showed highest accuracy among other pairs. QDA classifier did not show rapid change of testing accuracy over increase of number of subjects. Therefore, it might be usable with more subjects without compromising accuracy significantly. In this study, 15 trials were conducted, but when increasing the number of subjects, number of trials also should be increased to maintain consistent accuracy.

**Table 3:** Comparison with other studies.

Author	Electrodes	Task	Classified feature(s)	Classifier	Accuracy (%)
Ong et al. [50]	32	VEP	Power spectral density (PSD)	kNN	83
Alyasseri et al. [51]	6	Mental task (counting)	Multi-objective flower pollination algorithm with wavelet transform (MOFPA-WT)	ANN	85.12
Falzon et al. [42]	32	VEP	Frequency components up to 5th harmonic	kNN	91.7
Koutras et al. [52]	56	Sleep	Time-Domain Descriptors (10), Frequency-Domain Descriptors (17), Wavelet Domain Descriptors (4)	kNN	95
Fukami et al. [43]	4	VEP	5 frequency components	Mahalanobis distance	95
Yang et al. [53]	4	Motor movement, imagery	Wavelet log-DCT (WLD)	Fisher’s Linear Discriminant	98.5
Kaewwit et al. [54]	4	Resting state	Combined ICA and AR	kNN	98.51
Arnau et al. [55]	32	Video stimulation	PSD	ANN	99
Thomas et al. [56]	19	Resting state	PSD	Mean correlation coefficients	99
Schetinin et al. [57]	64	Motor movement, imagery	Group Method of Data Handling (GMDH)	SVM	100
Proposed approach	4	Relaxation before stimulation	IHAR	QDA, SVM, kNN	100
	4	Visual stimulation	IHAR	kNN	98.8 ± 0.9
	4	Mental recall	IHAR	QDA, SVM, kNN	100

Comparing the trial phases considered here, relaxation and mental recall showed higher accuracy. Selecting the simplest task among these three phases is complicated because there are merits and demerits for each. It is harder to concentrate or be relaxed in noisy environments, but visual stimulation would be easy. To stimulate the brain externally, some physical resource is needed, but other tasks can be performed spontaneously. In our approach, the relaxation phase showed highest accuracy for the minimal (four) electrode setup.

Mental recall of a number can be realized in many ways, including visualization (imagining an image of the number), audiation or silent verbalization (thinking of a sound, such as a voice reciting the number), synesthetic association (such as association of digits or numbers with colors, flavors, musical notes, etc.) [58] or cross-modal correspondences, cardinal correspondences (such as thinking of sets enumerated by a number’s digits), and spatial analogy (such as clock hour directions).

For an n-digit integer, *n* ≥ 2, the likelihood of a repeated digit is

$$\begin{array}{c} p\left(n\right)=1-\prod_{i=1}^{n-1}\frac{\left(10-i\right)}{10}. \end{array}$$ (15)

For a random four-digit integer, the probability is almost half that at least one digit is repeated, and such a pattern could anchor its recall. All of these recall styles are possible practices, and the actual technique was left up to each subject. This vagueness could explain apparent uselessness of data from the mental recall phase.

A major problem in EEG-based biometric systems is instability of signal patterns over time. EEG patterns can change with environment, maturity, and psychological disorders [59]. This issue has not been addressed in the proposed approach. Therefore, accuracy can be degraded over considerable time period due to changing daily mental states. Even though relaxation phase has been used as refactory period in this study, merits and demerits of taking off headset cannot be addressed with this experimental setup. In addition, results can be changed with different headsets because of hardware characteristics (resolution, sampling rate, common-mode rejection ratio) and type of electrodes.

IHAR was tested with different MA windows sizes (*L*) No substantial differences were found for different parameter values, but comparatively better results were obtained for *L* = 32. As *L* decreases, the size of ripples in the frequency response increases. As shown in Fig 7, even when *L* = 32, a series of ripples is produced, although the height of the ripples is not considerable.

## 3 Conclusion

In this research, we successfully deployed a novel feature, Inter-Hemispheric Amplitude Ratio (IHAR), to reveal personally unique information and distinguish people using EEG signals. With the proposed feature, highest accuracy could be achieved with a lower number of electrodes for a relatively simple task. Furthermore, this approach outperforms similar approaches with less computational power, suggesting deployment in portable devices with embedded low-power microprocessors. These promising results show that this approach has practical real-world applications. Also, this approach provides more convenience when training the system because there is no need of collecting large number of training trials. In the proposed approach, highest accuracy was achieved for the two frontal pairs of electrodes—FC5-FC6 and F7-F8. As a task phase to collect EEG data, relaxation is the best according to the results. Analysis showed the most suitable ML algorithms for classification are QDA, linear SVM, quatratic SVM, Gaussian SVM, and kNN. Moreover, AF3, AF4, F7, and F8 selective electrode arrangement can be used to develop tighter hardware design with high performance. However, accuracy would be affected if number of subjects increases. Also, brain signal patterns can significantly change over time, which would affect performance. For continued study, this approach can be further examined with a clinical-grade EEG system, and should be investigated to check whether the number of electrodes can be further reduced.

## Supporting information

S1 File (PDF)

S1 Dataset (ZIP)
