# Supplementary material for: Person identification from EEG using various machine learning techniques with inter-hemispheric amplitude ratio
Source: PLoS One. 2020 Sep 11;15(9):e0238872. doi: 10.1371/journal.pone.0238872 (PMC7485780; doi:10.1371/journal.pone.0238872)
Supplement: S1 File — (PDF) [file pone.0238872.s001.pdf]

Consent form

Research title: EEG-based biometric authentication system

Dear Sir/Madam,

Thank you for agreeing to be a part of our research. This research is conducted by the Department of Computing and Information Systems, Faculty of Applied Science.

EEG signals (simply brain signals) are used especially in the medical field to diagnose brain related illnesses. However, nowadays, this technology is getting popular in the computer science field because of the consumer-grade capturing devices. In this experiment, EEG signals are recorded for specific stimulation using an EEG headset with 14 electrodes. According to medical experts, this capturing process doesn't have any side effects to your brain or the body. The data collection is done to check the feasibility of EEG signals as biometric. After collecting EEG data, comprehensive analysis will be done using machine learning algorithms to investigate unique features of EEG signals to use as biometric.

We assure that your data will be kept anonymous and will not misuse. The collected data will be published with anonymous labels with the research work.

With regards,

Dr. Senaka Amarakeerthi.
Head of the Department,
Department of Computing and Information Systems,
Faculty of Applied Science,
Sabaragamuwa University of Sri Lanka.

Consent

I, ____ (Name) understand the nature of the data collection process and the research which is willing to perform. I Hereby give consent for recording my brain signals using an EEG headset to perform an analysis and publish them anonymously with the results of the research.

---------------------------------- ----------------------------

Signature Date

Questionnaire
(Circle your answer)

1) Age group: 20 - 24 / 25 - 29 / 30 - 34 / 35 - 39 / 40 - 44 / 45 - 50
2) Gender: Male / Female
3) Handedness: Left / Right
4) Do you have any mental illness (according to your knowledge)?: Yes / No
5) Are you taking any medicine for any of your illness?: Yes / No
6) Do you have any mental stress recently?: Yes / No
